# Flux Balance Analysis of Ammonia Assimilation Network in *E. coli* Predicts Preferred Regulation Point

**DOI:** 10.1371/journal.pone.0016362

**Published:** 2011-01-25

**Authors:** Lu Wang, Luhua Lai, Qi Ouyang, Chao Tang

**Affiliations:** 1 School of Physics, Peking University, Beijing, China; 2 Center for Theoretical Biology, Peking University, Beijing, China; 3 College of Chemistry and Molecular Engineering, Peking University, Beijing, China; 4 Department of Physics, Hong Kong Baptist University, Hong Kong, China; 5 Department of Bioengineering and Therapeutic Sciences, University of California San Francisco, San Francisco, California, United States of America; University of Pittsburgh, United States

## Abstract

Nitrogen assimilation is a critical biological process for the synthesis of biomolecules in *Escherichia coli.* The central ammonium assimilation network in *E. coli* converts carbon skeleton α-ketoglutarate and ammonium into glutamate and glutamine, which further serve as nitrogen donors for nitrogen metabolism in the cell. This reaction network involves three enzymes: glutamate dehydrogenase (GDH), glutamine synthetase (GS) and glutamate synthase (GOGAT). In minimal media, *E. coli* tries to maintain an optimal growth rate by regulating the activity of the enzymes to match the availability of the external ammonia. The molecular mechanism and the strategy of the regulation in this network have been the research topics for many investigators. In this paper, we develop a flux balance model for the nitrogen metabolism, taking into account of the cellular composition and biosynthetic requirements for nitrogen. The model agrees well with known experimental results. Specifically, it reproduces all the ^15^N isotope labeling experiments in the wild type and the two mutant (ΔGDH and ΔGOGAT) strains of *E. coli*. Furthermore, the predicted catalytic activities of GDH, GS and GOGAT in different ammonium concentrations and growth rates for the wild type, ΔGDH and ΔGOGAT strains agree well with the enzyme concentrations obtained from western blots. Based on this flux balance model, we show that GS is the preferred regulation point among the three enzymes in the nitrogen assimilation network. Our analysis reveals the pattern of regulation in this central and highly regulated network, thus providing insights into the regulation strategy adopted by the bacteria. Our model and methods may also be useful in future investigations in this and other networks.

## Introduction

**Figure 1:**
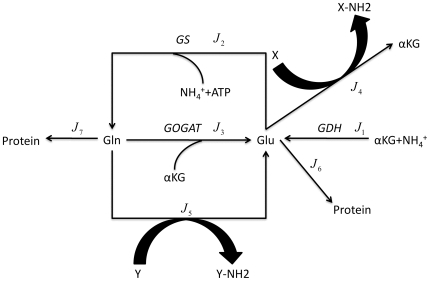
The schematic model of the nitrogen assimilation network. Arrows denote the direction of the reactions. *GDH*, *GS* and *GOGAT* denote the enzymes catalyzing the reactions. For GDH, one αKG and one NH_4_^+^ are converted to one Glu. And for each turn of GS-GOGAT cycle, one more ATP is needed to form one Glu. X and Y denote all other nitrogen-containing metabolites obtaining their nitrogen atoms via Glu- or Gln-dependent aminotransferases, respectively.

For *Escherichia coli*, ammonia is the preferred nitrogen source that supports its fastest growth [1]. The first step in ammonia assimilation is the synthesis of glutamate (Glu) and glutamine (Gln). As shown in Fig. 1, there are two pathways dedicated to this step in *E. coli*. One pathway involves the NADP-linked glutamate dehydrogenase (GDH, EC 1.4.1.4), which converts ammonium and α-ketoglutarate (αKG) to glutamate. The other pathway involves the combined activities of the glutamine synthetase (GS, EC 6.3.1.2), which aminates glutamate to form glutamine, and the glutamate synthase (GOGAT, EC 1.4.1.13), which transfers the amide group from glutamine to αKG to produce two molecules of glutamate [1], [2]. The nitrogen atoms in almost all nitrogen-containing metabolites in *E. coli* are derived from glutamate and glutamine, the two primary products of ammonium assimilation [3]. In particular, these two amino acids provide nitrogen for all other amino acids and the nucleotides. Glu directly or indirectly provides α-amino groups for most of the 20 amino acids and around half of the nitrogen for pyrimidine, purine and the amino group of adenine (see Table S1) [4], [5]. Gln provides the remaining nitrogen supply for purine and pyrimidine, and the nitrogen for asparagine, histidine and tryptophan (see Table S1) [4], [5].

Experimental observations on bacteria growth suggested that *E. coli* tend to maintain an optimal growth under a wide range of the external ammonia concentration [6]. This presumably implies that in response to different ammonia availability the ammonia assimilation network is regulated in such a way as to maintain a right distribution of nitrogen fluxes to a variety of metabolites [1]. An important question is: what is the regulation strategy.

Since Stadtman's pioneer work in the late 1970s [7], [8], [9], some theoretical work has focused on the elaborated and detailed regulation on GS and analyzed the complex interplay between covalent modification cycles and allosteric interactions [10], [11]. Later work moved onto establishing ordinary differential equation (ODE) models and simulating the systemic dynamics [12], [13], [14], [15], [16]. More recently, Yuan and coworkers combined their ODE model with massive experimental data of metabolomics to investigate the hypothesis of active-site competition on GOGAT [17]. These work and models focused on specific questions of regulation and studied the system behavior in different conditions. However, the overall picture of the regulation, especially the link between the regulation points and the bacteria growth, is still not clear. In this work, we develop a metabolic flux balance model based on the fundamental biological data, linking the nitrogen flux requirement for growth to the regulation of the ammonia assimilation network. The model is used to calculate the stationary flux distributions and the dynamics of ^15^N isotope labeling process for the wild type and mutation strains. The results agree well with the isotope labeling experiments [17], [18]. Furthermore, using the catalytic reaction equations of GDH, GS and GOGAT, we predict their V_max_ values in different growth conditions, which are also found to be consistent with experimental observations [17]. Finally, based on this flux balance model and the principle of minimal regulation, we demonstrate the rationality of GS as the preferred regulation point among the three enzymes in the nitrogen assimilation network.

## Results

### Ammonium Diffusion across the Membrane and Ionization Equilibrium

The nitrogen assimilation process of *E. coli* starts from the ammonium (NH_4_^+^ + NH_3_) diffusion across the cellular membrane. However, only the uncharged NH_3_ can diffuse freely through the membrane with a high permeability [6], [19], [20], [21], [22]. Since the pK_a_ of NH_4_^+^ is 9.25, external NH_3_ concentration (NH_3_ex) is relatively low: about 55.92 µM at pH 7 when total ammonium (NH_3_ex + NH_4_^+^ex) is 10 mM. Besides the free diffusion of neutral ammonia, *E. coli* can transport ammonium (NH_4_^+^ex) by its transporter protein AmtB [23], [24], [25]. However, due to the estimated density (10 to 1000 per µm^2^) and transporting efficiency (10 to 10^4^ ammonium per second per transporter) [26], it only functions in a very low ammonium level or low pH environment [6]. After NH_3_ex diffuses into the cytoplasm, internal NH_3_ (NH_3_in) is protonated into NH_4_^+^in, which serves as the substrate of GDH and GS [27], [28]. The permeation of NH_3_ can be described by
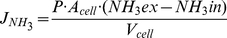
(1)where 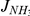 denotes the ammonia assimilation flux, 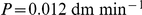
is the permeability coefficient [20], [22], 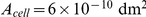
is the surface area of *E. coli* cells [29], and 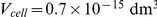 is the cellular volume (personal communication with Dr. Yuan ).

### Metabolite Flux Distribution for Wild Type Cells

As shown in several experiments, the cell mass of *E. coli* exponentially increases with the growth rate, and the cellular volume increases with a similar speed as the cell mass [30], [31]. This means that the concentrations of internal metabolites and the mass flux per unit volume are better quantities to monitor in our work. We used mM and mM/min as the units of concentration and flux in the following. The metabolic system of nitrogen assimilation outlined in Fig. 1 contains GDH, GS, and GOGAT catalytic reactions (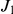, 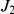, and 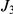), Glu- and Gln-dependent aminotransferase reactions (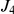 and 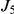), and the consumption of Glu and Gln as the metabolic carbon skeleton or protein residues (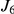 and 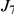). During the exponential growth phase, the fluxes and the concentrations of Glu and Gln in our system are assumed to be constant [32]. Then, following the law of mass conservation, we have
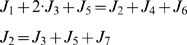
(2)


To obtain the respective contribution of Glu and Gln to aminotransferase reactions and as the carbon skeleton, we used the cellular composition and biosynthetic requirements for nitrogen in *E. coli* from Table 2 in Ref. [33] and calculated the details of the nitrogen donor for all compounds in that table. The result is summarized in Table S1. At the same time, we obtained the cellular volume (about 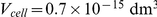) and cell dry weight (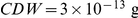) from the footnote of the same table to rescale the unit from mass amount per gCDW to mM. We then derived our fluxes 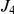, 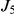, 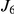 and 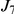
by dividing the concentrations with the doubling time which can be collected from the experimental works. The results are listed in Table 1. The rest of the variables of the 7-variable Eq. (2) can be estimated as the following. Since GS is the only reaction to synthesize Gln in *E. coli*, the input of synthesizing flux equals to the output of consuming flux. Therefore we used the measured consuming flux of Gln directly taken from Table 1 of [18] as the flux of GS: 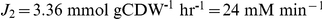. With the above five fluxes estimated from experimental measurements, we solved the algebraic equations to obtain the other two fluxes: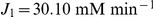 and 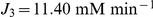. The total ammonium consumption flux 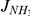 equals to the summation of 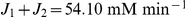. Considering the concentration of the external ammonium to be 10 mM as in [18], we got 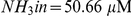 and protonated 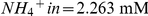 from Eq. (1) and the ionization constant of NH_3_, using pH = 7 in the medium and pH = 7.6 inside the cell [2], [34], [35].

**Table 1:** Nitrogen contribution from Glu and Gln at doubling time = 80 minutes.

	N donation		C skeleton	
	Glu (*J*_4_)	Gln (*J*_5_)	Glu (*J*_6_)	Gln (*J*_7_)
Total (mmol g_CDW_^−1^)	6.7274	2.1024	0.7686	0.250
Flux (mM min^−1^)^*^	36.04	11.26	4.118	1.339

**Table 2:** Prediction of V_max_ at 3 hours grown in 2mM ammonium.

Experimental conditions	WT (10mM)	WT (2mM 3h)	ΔGDH (2mM 3h)	ΔGOGAT (2mM 3h)	WT/ΔGDH (2mM 3h)	WT/ΔGOGAT (2mM 3h)
Doubling time (min)	57	110	110	110	-	-
NH_4_^+^ex + NH_3_ex (mM)	10	0.75	0.75	0.75	-	-
NH_4_^+^in (µM)	2174	16.47	16.47	16.47	-	-
Glu (mM)	96	76.56	78.89	43.45	-	-
Gln (mM)	3.8	1.95	2.067	12.97	-	-
αKG (mM)	0.375	11.65	9.59	15.47	-	-
J_1_ (mM/min)	20.62	11.36	0	30.18	-	1:2.66
J_2_ (mM/min)	54	27.98	30.20	9.17	1:1.0793	1:0.328
J_3_ (mM/min)	36.62	18.82	39.34	0	1:2.0903	-
J_1_/( J_1_+ J_2_)	27.6%	28.9%	0	76.7%		
V_max_ of GDH (mM/min)	413	1267	0	3012	-	1:2.38
V_max_ of GS (mM/min)	649	2306	3240	1082	1:1.405	1:0.469
V_max_ of GOGAT (mM/min)	63.79	33.00	52.29	0	1:1.5845	-

### Verification of the Flux Distribution by Experiments for Wild Type and Knockout Strains

**Figure 2:**
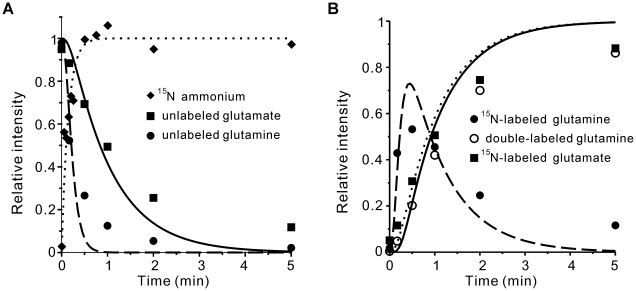
Isotope labeling kinetics of the central intermediates in nitrogen assimilation. (A) Curves represent the model simulations of the decay kinetics for the unlabeled glutamate (solid line) and the unlabeled glutamine (dashed line), and of the ammonia diffusion kinetics (dot line). Symbols represent the experimental data from (Yuan et al, 2006). (B) Curves represent the model simulations of the labeling kinetics for the formation of ^15^N-labeled glutamate (dot line), single-labeled glutamine (dashed line) and double-labeled glutamine (solid line). Symbols represent the experimental data from (Yuan et al, 2006).

We verified the flux distribution using the *in vivo* experimental data reported by Yuan in 2006 [18]. The experimental procedure is the following: when cells in the exponential growth phase were switched from unlabeled to ^15^N isotope-labeled ammonium, the ammonium in the medium and the nitrogen in the intracellular metabolites, such as amino acids and nucleotides, can be traced. The dynamics process can thus be recorded. With the above obtained data of fluxes and the concentrations of the external ammonia and NH_4_^+^in, and taking the concentrations of Glu and Gln as the values measured in [18], this process can be simulated under the assumptions that (1), during the shift process, the total concentrations of the external ammonia and internal metabolites remain unchanged, and (2), the entire flux distribution remains unchanged [18]. Details of the ordinary differential equations can be found in File S1. As shown in Fig. 2, our parameter-free simulation catches the essential dynamic features of the Glu and Gln fluxes. Overall, the predicted labeling kinetics of cytoplasmic ammonia, Glu and Gln (Fig. 2A), and the kinetics of Glu synthesis (either directly from ammonia via GDH or indirectly via GS-GOGAT) and of Gln synthesis (Fig. 2B) match the experimental data. We found that the quantitative discrepancies between experiments and simulation mainly come from one source: the concentrations of metabolites (external ammonium, internal Glu and Gln). If we allowed a fine-tuning of these parameters, we can quantitatively fit the experimental data (see the simulation shown in Fig. S1 in Supporting Information, which we only changed one parameter, the concentration of Gln).

To further validate our model, we also investigated the labeling process of two mutant strains, GDH knockout (ΔGDH) and GOGAT knockout (ΔGOGAT). Since the growth rate of both knockout strains are almost the same as that of the wild type in 10 mM and 2 mM ammonium concentrations (see the experiment details in [17]), we assumed that the cellular composition and biosynthetic requirements for nitrogen are the same for the wild type and the two mutant strains. We first solved the algebraic equations of flux balance (Eq. (2)) using the doubling times (58 min, 56 min and 57 min) taken from the Supplemental Table 1 of Yuan's work [17] for the wild type and the two mutants. For the two mutants (ΔGDH and ΔGOGAT), Eq. (2) have 6 variables; it could be solved without the information of the measured Gln flux.

Table 2 summarizes the calculation result. Both GDH and GS can assimilate ammonium into the metabolic network, but they cannot substitute each other. GS-GOGAT cycle costs one ATP for every Glu formed, while ATP is not used in the GDH reaction. However, the K_m_ for ammonium of GDH (about 1.1 mM) is much higher than that of GS (0.1 mM) [27], [28]. Thus, their contribution to ammonium assimilation should be different under different situations. Indeed, from our calculations, the ratio of 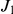 to total ammonium assimilation flux (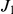
+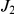) changed from 27.6% for the wild type to 76.7% for the ΔGOGAT strain (Table 2). The flux distributions of the wild type and the two mutants are shown in Table S2. The predicted flux of GS 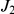 for ΔGDH (77.28 mM/min) and ΔGOGAT (17.69 mM/min) is consistent with the measured fluxes for ΔGDH (57±26 mM/min) and ΔGOGAT (13±2 mM/min) taken from Supplementary Table 1 of [17]. The network of ΔGDH increased both 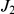 and 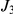 to compensate the effect of missing GDH, which also agree with the experimental observation [2], [36]. For the ΔGOGAT strain, it only has the linear GDH-GS pathway to synthesize Glu and Gln. Our results showed about 3-fold changes of 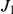 increase and 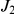 decrease, which again agreed with the observations [37].

**Figure 3:**
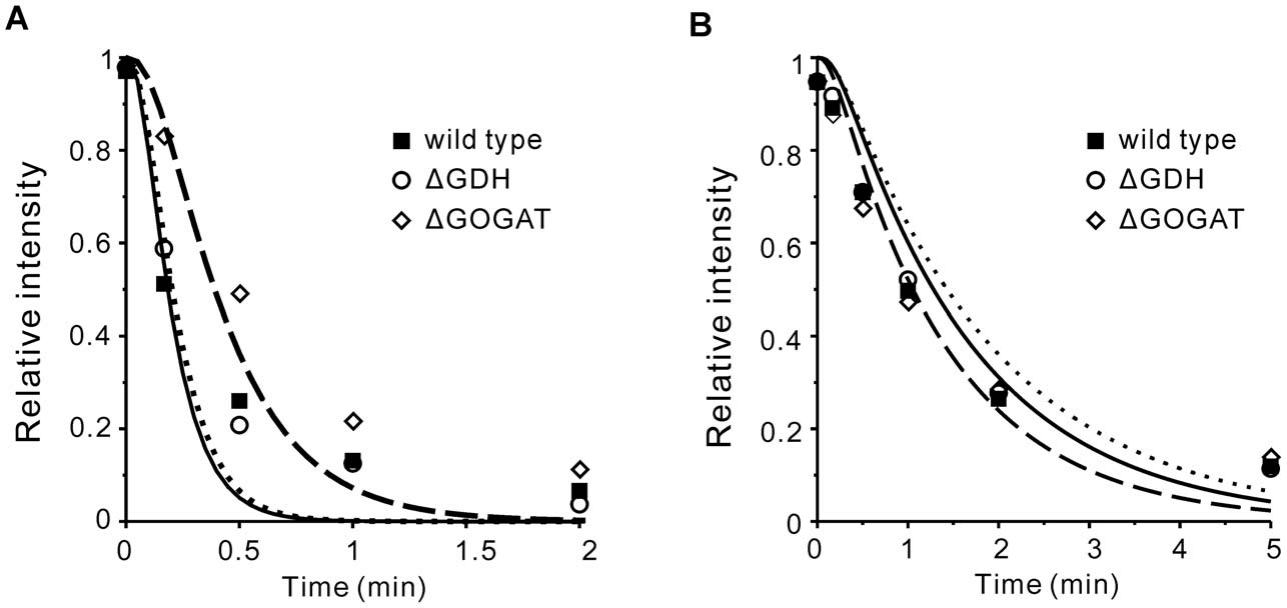
Isotope labeling kinetics of the wild type, ΔGDH and ΔGOGAT strains. (A) Decay kinetics of unlabeled glutamine. (B) Decay kinetics of unlabeled glutamate. In both A and B, curves represent the model simulation for the wild type (dot line), ΔGDH (solid line) and ΔGOGAT (dashed line). Symbols represent the experimental data from (Yuan et al, 2009).

Next, based on the flux distribution in Table S2, the nitrogen atom labeling process for ΔGDH and ΔGOGAT strains was studied by using the same method described above. The concentrations of Glu and Gln were obtained from Supplemental Table 1 of [17], and the concentrations of NH_4_^+^in were estimated using Eq. (1) for the wild type, ΔGDH and ΔGOGAT strains. As shown in Fig. 3B, the calculated kinetics of labeling Glu perfectly matches the experimental results. Because the ΔGOGAT strain breaks the GS-GOGAT cyclic pathway and synthesizes Glu only through GDH, the decrease of Gln consumption flux induced a decrease of the GS flux and slowed down the Gln labeling kinetics compared with the wild type and the ΔGDH strains (Fig. 3A). However, the labeling kinetics of Gln in the wild type and the ΔGDH strains are similar. These results also agree well with the experimental observations [17].

### Detailed Chemical Reactions and Prediction of Enzyme Activities for ΔGDH and ΔGOGAT Strains

The experimental validations gave us confidence in our flux balance model. However, it does not provide any information about the details of the regulation on the enzymes involved in the model. To investigate the regulation details in the nitrogen assimilation network, we employed the kinetic equations built by Bruggeman and coworkers to model the fluxes of the reactions catalyzed by GDH (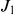), GS (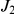) and GOGAT (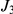) (see Eq. (3) in Methods for details) [15].

In Eq. (3), GDH and GS reactions were considered as reversible and GOGAT reaction as almost irreversible [15], [17]. Besides the reaction constants (*K*, *L* and *M*), let us assume that the concentrations of the energy related metabolites (ATP, ADP, NADP, NADPH) are constants (or change little in the experimental conditions we consider below). Eq. (3) gives the fluxes of the enzymatic reactions as functions of the environment (NH_4_^+^in), the concentrations of substrates (αKG, Glu, Gln), and V_max_. Using the substrate concentrations measured in [17] and the flux values obtained from the analysis in the previous section, we can calculate the V_max_ for various experimental conditions. We did this for two cases in which there were experimental data that can be used to estimate V_max_: (1) the samples grown in 10 mM ammonium in the exponential growth phase, and (2) the samples obtained 3 hours after the cells grew on 2mM ammonium plates to nitrogen limitation, for the wild type and the mutant strains of ΔGDH and ΔGOGAT, respectively (see the experimental detail described in [17]). Table 2 summarizes the results of our calculation for the two cases (the details of the calculation are in File S2). If we assume that the V_max_ value of an enzyme is proportional to the enzyme protein level, then our results agree well with the experiments – our calculated V_maxs_ matches quite well with the protein level obtained from the western blot of the enzyme in the same condition. For GS, our calculation shows that the V_max_ values of the wild type and the ΔGDH strains grown in 2 mM ammonium are higher than that of the wild type in 10 mM ammonium. And the V_max_ of the wild type strain grown in 2 mM ammonium is just 2 times higher than that of the ΔGOGAT strain. These predictions were confirmed by the western blot data shown in Fig. 2C of Yuan's work [17]. For GDH, the V_max_ behaves differently than that of GS. Our calculation shows that the ΔGOGAT strain has the highest level of V_max_, because it needs to compensate the missing synthesis pathway of Glu through GOGAT. Its level is 2.38 folds of the wild type strain in 2 mM ammonium, and the level of the wild type strain in 10 mM ammonium is lower than that in 2 mM ammonium. These predictions are also in good agreement with the western blot data [17]. For GOGAT, our calculation shows that the V_max_ for the wild type strain in 10 mM ammonium is about 50% of the value for cells grown in 2 mM ammonium. For the ΔGDH strain, the V_max_ almost recovers to the level of the wild type. To sum up, among different strains and different conditions, the V_max_ values of GDH and GS have about 5–7 folds change, and V_max_ of GOGAT varies in a much narrower range. This seems to suggest that GDH and GS are regulated more than GOGAT [1], [37], [38].

### Preferred Regulation Point in Nitrogen Assimilation Network

When grown in a minimal medium, *E. coli* was observed to maintain a relatively constant doubling time (about 60 min) in a wide range of the external ammonium concentration [6], [17], [18], [39]. To achieve the same growth rate in different ammonia concentrations, it is reasonable to assume that the fluxes of 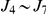 are unchanged since these fluxes are directly related to the rates of biosynthesis of proteins, nuclear acids, and other biomolecules that together form the biomass. Hence, there must be some regulations on the nitrogen assimilation network to keep these fluxes constant under varied external conditions. What would be the most efficient way of regulation in order to keep these fluxes constant? We now address this question within the framework of Eqs. (2) and (3).

In principle, a global control that involves regulating each and all of the fluxes in ammonium assimilation can result in constant 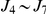. In this scenario, presumably many enzymes would have to be regulated separately. Here we consider another scenario that involves regulating only the three major enzymes GDH, GS and GOGAT (Fig. 1). It is conceivable that *E. coli* would prefer a strategy of using fewer regulations to achieve the same objective, assuming everything else being equal. Even if in reality more enzymes are being regulated in the regime of nitrogen availability we consider, investigating the capability of the regulation on the three major enzymes towards maintaining a constant growth rate would still be illuminating [4].

Using the wild-type values of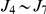 from Table S2 in Supporting Information as the constant flux values for the constant growth, the two mass conservation equations (1) are left with three undetermined fluxes 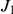, 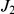 and 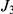. Substituting the kinetic equations (3) for the three fluxes, we obtain a system of two equations relating nitrogen availability (NH_4_^+^in) with V_maxGDH_, V_maxGS_, V_maxGOGAT_. For a changing NH_4_^+^in concentration, one can find corresponding changes in these V_max_'s, which would reflect the regulations on the respective enzymes (GDH, GS and GOGAT) to maintain the constant growth rate. However, there are many more variables than equations in this system. Certain assumptions are needed to confine the solution space of the V_max_'s. We assume that the energy related metabolites (ATP, ADP, NADP and NADPH) do not change significantly under the nitrogen limitation conditions we are considering. Among the three substrates Gln, Glu and αKG, Glu was observed to stay at a constant high level to maintain the internal pool of K^+^, the most prevalent osmolyte inside the cell [37], [39], [40]. On the other hand, both Gln and αKG can vary with the external ammonium concentration and the growth rate [39], [41]. With the assumption of constant energy metabolites and Glu, we are left with 5 variables (V_maxGDH_, V_maxGS_, V_maxGOGAT_, Gln and αKG) that should satisfy the two equations of mass conservation. Since the system is still under-determined, we proceeded with the following two approaches.

**Figure 4:**
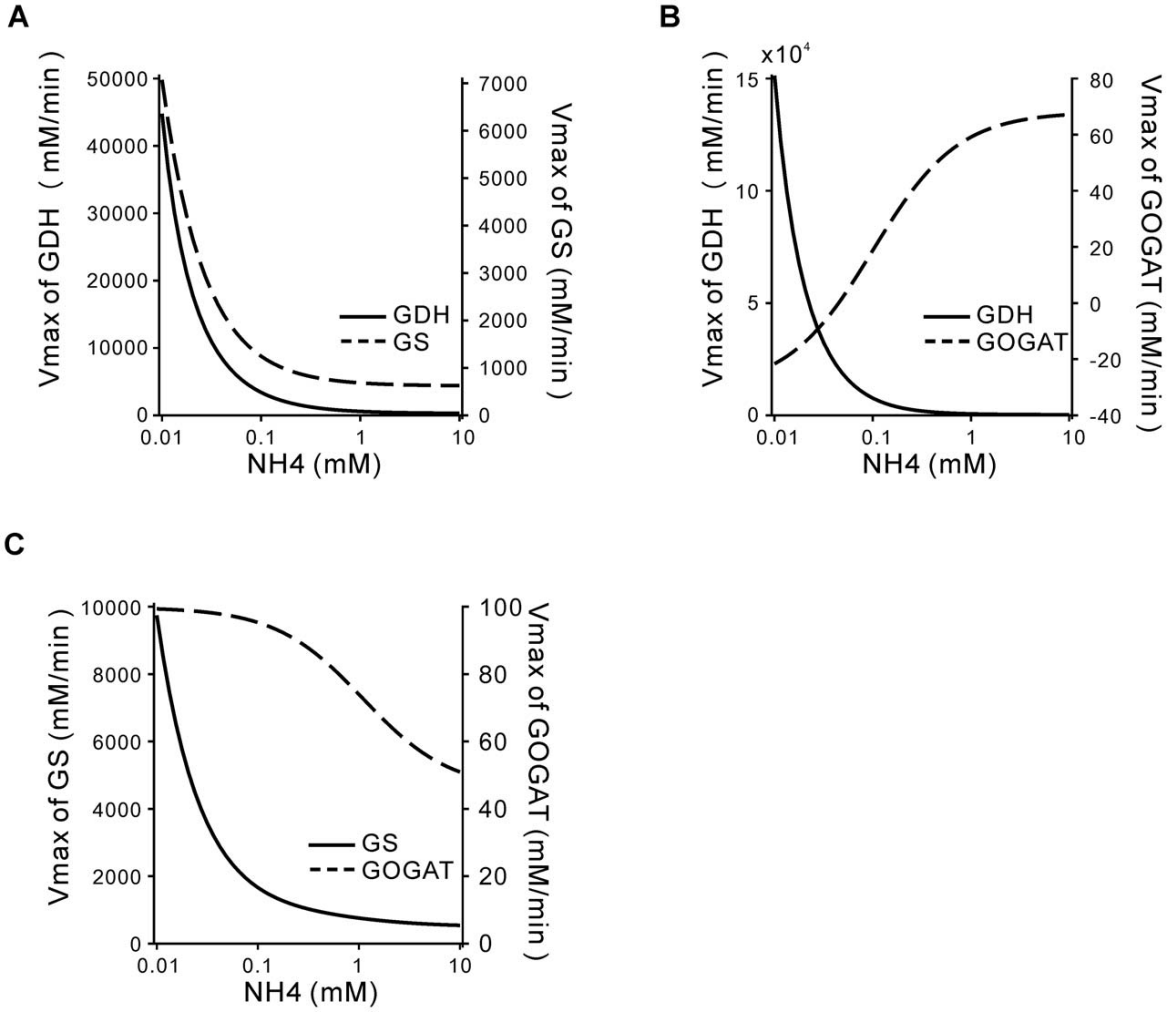
Response curves for pairs of enzymes to achieve the objective function against changes in the external ammonium concentration. (A) V_max_ of GDH and GS. (B) V_max_ of GDH and GOGAT. (C) V_max_ of GS and GOGAT.

We first let two of the V_max_'s to vary in response to the changing ammonia concentration, and kept the other three variables fixed. (The fixed variables take the values under ammonia rich conditions, i.e. the first column of Table 2). There are three combinations of two V_max_'s: GDH-GS, GDH-GOGAT and GS-GOGAT. Their response curves with changing external ammonia availability are shown in Fig. 4. For the combination of GDH and GS, the V_max_ of GOGAT was fixed at 63.79 mM/min (Table 2). The result shows that in this case in order to achieve the regulation goal against a variation of NH_4_^+^ from 10 mM to 0.01 mM, the V_max_ of GDH has to vary about 150-fold (from 304 to 44780 mM/min), and the variation of the V_max_ for GS also needs to exceed 10-fold (from 607 to 7070 mM/min) (Fig. 4A). For the combination of GDH and GOGAT, the V_max_ of GS was fixed at 649 mM/min. The curve of the V_max_ of GDH with changing NH_4_^+^ shows that its variation was nearly 550-fold (from 276 to 151258 mM/min) (Fig. 4B). When NH_4_^+^ decreased below about 0.05 mM, the V_max_ of GOGAT became negative. Since this reaction is strongly forward driven, GOGAT functioning on the reverse direction was unreasonable [15], [17]. For the combination of GS and GOGAT, the V_max_ of GDH was fixed at 413 mM/min. The result shows that the V_max_ of GOGAT only need to change about one fold. And the variation of the V_max_ for GS was about 18-fold (from 541 to 9743 mM/min) (Fig. 4C). To sum up, the combination of GDH and GOGAT can be the first to rule out. For the combination of GDH and GS, because GDH is usually high for *E. coli* grown in glucose-ammonia minimal medium and plays an important role during glucose-limited growth [2], [36], [42], [43], regulating its catalytic activity in 150-fold range is a hard task comparing with approximately 7-fold change of GDH V_max_ predicted in the last section. In contrast, the last combination of GS and GOGAT only required 18-fold variation of the GS V_max_ and one-fold for the GOGAT V_max_. Actually, GS enzyme is a dodecamer of identical 55000-Da subunits. Each subunit can be adenylylated to impair its own catalytic activity [44], [45], and its transcriptional level is also finely regulated in a multifold range by the NRI-NRII two-component system [39], [46], [47]. Therefore, it seems reasonable that the activity of GS can be regulated in tens of folds, which was indeed observed in experiments [41], [48].

**Figure 5:**
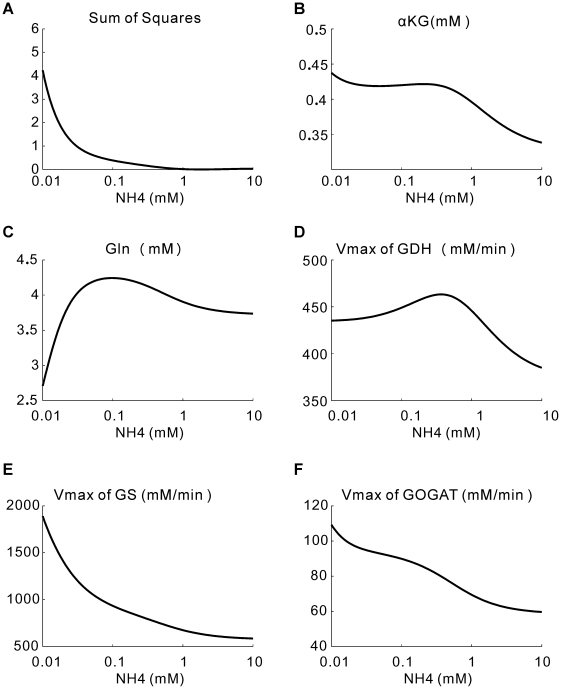
Minimal solutions of the 5 variables in response to NH_4_ changes. The x-axis is the NH_4_ concentration inside of the cell. (A) The sum of squared relative changes of the 5 variables. (B) αKG. (C) Gln. (D) V_max_ of GDH. (E) V_max_ of GS. (F) V_max_ of GOGAT.

Next, we let all the 5 variables (V_maxGDH_, V_maxGS_, V_maxGOGAT_, Gln and αKG) vary in response to a changing ammonium concentration. There will be infinitely many solutions. We focus on the “minimal solution” for each ammonium concentration. A minimal solution is the one that minimizes the summed changes of the 5 variables. We searched for the minimal solutions corresponding to different ammonium concentrations that minimized the squared distance Z from their original reference values (Fig. 5; see Methods for the details). We tried both local and global searches, and both gave the same results. The results of the minimal solutions are shown in Fig. 5. The squared difference Z from the reference maintained at low values for internal ammonium concentrations higher than 0.1 mM (Fig. 5A), indicating that small changes in the activities of the enzymes are sufficient to cope with changes of the ammonium level within this range. When the ammonium concentration falls below 0.1 mM, Z increases rapidly, suggesting significant regulation of enzyme activities in this region. Nonetheless, despite 1000-fold change of the internal ammonium concentration, these variables showed relatively small changes except for the GS activity (Fig. 5E).

Taken together, these results suggest that GS would serve as a main point of regulation in the ammonia assimilation network. It is well-known that GS is a major regulation point in this system [49]. Here we identified it as the preferred regulation point using our flux balance model only, without any other prior information on the enzymes.

## Discussion

Several simulation works have been conducted on the *E. coli* ammonia assimilation network in recent years [14], [15], [16], [17]. While insights were gained from these studies, it remains a challenge to comprehend the massive amounts of experimental data accumulated since decades ago. One reason is that the three central reactions in the nitrogen assimilation network are intensely coupled with the ammonium uptake through the physiological metabolism of glutamate and glutamine, and with the TCA cycle that supplies and consumes the carbon-skeleton component αKG. It is difficult to simulate the proper boundary conditions *in vivo*. Another reason for the limited progress in modeling this system is that the nitrogen assimilation system consists of a complicated network of interactions amongst proteins, genes and small molecules. Although in recent years many proteins involved in this system have been characterized in detail [50], [51], [52], [53], [54], the research on the dynamics of the regulation network still needs many kinetic parameters, and many transient time-course data to calibrate and validate the model. This motivated us to take an alternative approach to study the system. In the first part of this paper, we presented a flux balance model based only on the fundamental metabolic data and the overall topological structure of the network. The model agrees well with the experiments on the kinetics of metabolites distribution in wild type and mutant cells. The model contained a few very simple assumptions and has no other adjustable parameters. Therefore it is easy to verify or falsify the model assumptions and predictions with more experimental data. In the second part of the paper, we considered how regulation of the enzyme activities in response to decreasing ammonia availability can help to achieve an optimal growth. Here we relied on more detailed flux equations (Eq. (3)) which contain kinetic parameters. Although these parameters were derived from extensive in vitro experiments, there is no guarantee that the equations are accurate in vivo. We would like to emphasize that our goal here is not to precisely predict the exact regulation of each enzymes. Rather, we want to get an overall picture of the regulation and the preferred regulation point(s). And for this purpose, our conclusions should not be very sensitive to the details of Eq. (3).

Several notes are in order. First, in our model the ammonium uptake process was considered as the neutral NH_3_ex free diffusion across the membrane and then protonation in the cytoplasm. Although the permeability coefficient for NH_3_ measured in different experimental conditions spanned over several orders of magnitude [6], [19], [20], [55], it did not qualitatively influence our results (data not shown). Our more physical-based model of NH3 diffusion also produced similar apparent diffusion parameters of the more phenomenology-based model used in [17].

Second, our model study suggested that besides GS, GOGAT may also be regulated when the growth environment changes. Previously almost all kinetic models focused only on the classic chemical and transcriptional regulation cascades on GS; the role of the regulation on GDH and GOGAT remains to be an open question. There were a number of experimental studies on GDH and GOGAT regulation [37], [56]. Our model may provide a useful guide in the future study of the regulation role of GDH and GOGAT.

Third, in our model, one important assumption was that the nitrogen composition and consumption distribution are kept the same under all growth conditions [32]. However, this is still a controversial issue [57]. For different growth rates, the cellular compositions such as DNA, RNA and proteins are different [3], [30], [58], [59]. It will be an interesting question to investigate how this composition difference influences the nitrogen consumption distribution and its consequence on the regulation of the nitrogen assimilation network.

Fourth, in response to environmental changes, the bacterial metabolic network redistributes the fluxes to optimize growth. For a metabolic network as complex as that in *E. coli*, there can be many different ways of regulation to achieve the same goal [60]. It is unclear if there exists some kind of general regulation strategies for the bacteria. It is conceivable that the bacteria may want to use a minimal effort/cost to achieve the objective. However, given the complexity of the network structure, pleiotropic constraints and the evolutionary history, it remains to be seen to what extend this is possible and how it is implemented. Our work on preferred regulation points may shed some light and stimulate further studies in this direction.

## Methods

### The catalytic reactions of GDH, GS and GOGAT

We adopt the following equations from Bruggeman and coworkers (Bruggeman *et al*, 2005):
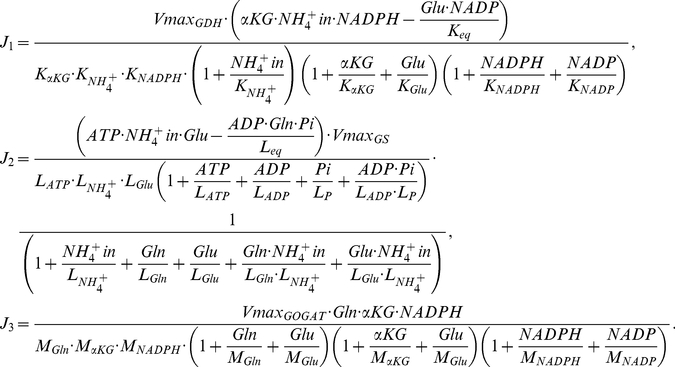
(3)where the V_max_ are the maximum reaction rates for GDH, GS and GOGAT, respectively; *K*, *L* and *M* represent the reaction constants involved in the reactions (see Table S3 in Supporting Information) ) [15]. The equations were derived based previous experimental researches on reaction mechanisms [27], [61], [62] and contained dozens of kinetic parameters. The values of these kinetic parameters came from decades of accumulation of *in vitro* experimental measurements, which were listed in Table S3 [15]. To our knowledge, these equations are the most detailed and reliable ones up to date.

### Minimal solutions

To search for the “minimal solution” in the variables' space, we defined the squared distance Z between two solutions as the sum of the square of the relative changes for all variables *X_i_*: 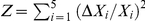. The reference values for the 5 variables are taken to be the ones under 10 mM external ammonium concentration (the first column in Table 2). In the local search, we varied the ammonium concentration gradually (from the starting condition which defines the reference values of the 5 variables) step by step and identified as the minimal solution in each step that minimized its squared distance Z with the previous solution. In the global search, we randomly chose the initial values of these 5 variables within the range between 1/10 and 10-fold of their reference values, and optimized these values for the given ammonium concentration by minimizing the squared distance Z from the reference values. For a given ammonium concentration, we repeated this process 3000 times with different initial values of the 5 variables. For the vast majority of the initial values (99%), the global search converged to the same minimal solutions obtained from the local search. The rest of the initial conditions did not converge to any meaningful solutions.

## Supporting Information

Figure S1 To improve the fitting to the isotope labeling dynamics of Gln and Glu, we kept the condition as the same as Fig. 2, except setting the concentration of Gln to 7 mM. (A) Curves represent the model simulation of the decaying kinetics of the unlabeled glutamate (solid line) and the unlabeled glutamine (dashed line). Symbols represent the experimental data from Yuan's work in 2006. (B) Curves represent the model simulation of the labeling kinetics for the formation of glutamate (dot line), single-labeled glutamine (dashed line) and double-labeled glutamine (solid line). Symbols represent the experimental data from Yuan's work in 2006.
(EPS)

Table S1 (DOC)

Table S2 (DOC)

Table S3 (DOC)

File S1 The ordinary differential equations describing the labeling process of the metabolites.
(DOC)

File S2 Predicted key enzyme activities for ΔGDH and ΔGOGAT Strains.
(DOC)
