# Supplementary material for: Flux Balance Analysis of Ammonia Assimilation Network in E. coli Predicts Preferred Regulation Point
Source: PLoS One. 2011 Jan 25;6(1):e16362. doi: 10.1371/journal.pone.0016362 (PMC3026816; doi:10.1371/journal.pone.0016362)
Supplement: Table S1 — (DOC) [file pone.0016362.s002.doc]

***Supplementary Table 1^a^***

| Residues | Composition | Total N atoms per molecule | N atoms from Glu | N atoms from Gln | N atoms from others |
| --- | --- | --- | --- | --- | --- |
| Protein amino acids |  |  |  |  |  |
| alanine | 488 | 1 | 1 |  |  |
| arginine | 281 | 4 | 3^b^ | 1 |  |
| asparagine | 229 | 2 | 1 | 1 |  |
| aspartate | 229 | 1 | 1 |  |  |
| cysteine | 87 | 1 | 1 |  |  |
| glutamate | 250 | 1 | 1^b^ |  |  |
| glutamine | 250 | 2 |  | 1^c^ |  |
| glycine | 582 | 1 | 1 |  |  |
| histidine | 90 | 3 | 2 | 1 |  |
| isoleucine | 276 | 1 | 1 |  |  |
| leucine | 428 | 1 | 1 |  |  |
| lysine | 326 | 2 | 2 |  |  |
| methionine | 146 | 1 | 1 |  |  |
| phenylalanine | 176 | 1 | 1 |  |  |
| proline | 210 | 1 | 1^b^ |  |  |
| serine | 205 | 1 | 1 |  |  |
| threonine | 241 | 1 | 1 |  |  |
| tryptophan | 54 | 2 | 1 | 1 |  |
| tyrosine | 131 | 1 | 1 |  |  |
| valine | 402 | 1 | 1 |  |  |
| RNA and DNA nucleotides |  |  |  |  |  |
| AMP and dAMP | 189.6 | 5 | 3 | 2 |  |
| GMP and dGMP | 228.4 | 5 | 2 | 3 |  |
| CMP and dCMP | 151.4 | 3 | 1 | 1 | 1^d^ |
| UMP and dTMP | 160.6 | 2 | 1 | 1 |  |
| Others |  |  |  |  |  |
| Glucosamine | 16.8 | 1 |  | 1^e^ |  |
| Ethanolamine | 122.2 | 1 | 1^f^ |  |  |
| N-Acetylglucosamine | 27.6 | 1 |  | 1^e^ |  |
| N-Acetylmuramic acid | 27.6 | 1 |  | 1^e^ |  |
| Alanine^g^ | 55.2 | 1 | 1 |  |  |
| diaminopimelate | 27.6 | 2 | 2^h^ |  |  |
| Glutamate^i^ | 27.6 | 1 | 1^b^ |  |  |
| Putrescine | 34.1 | 2 | 2 |  |  |
| Spermidine | 7.0 | 3 | 3 |  |  |

^a^ the residue composition data adopted from Table 2 in Ref. [1]. Most of the data of nitrogen donor for each residue were collected from CHAPTER 3 “Biosynthesis of Escherichia coli cells from Glucose” in Ref. [2] and CHAPTER 9 “Metabolism of lipids, nucleotides, amino acids, and hydrocarbons” in Ref. [3].

^b^ Glutamate serves as carbon skeleton.

^c^ Glutamine serves as carbon skeleton.

^d^ this one nitrogen atom comes from ammonia directly

^e^ see http://biocyc.org

^f^ its nitrogen comes from serine directly.

^g^ it represents the amount of alanine in peptidoglycan components

^h^ its nitrogens come from lysine directly.

^I^ it represents the amount of Glutamate in peptidoglycan components

**References**
