# Supplementary material for: Flux Balance Analysis of Ammonia Assimilation Network in E. coli Predicts Preferred Regulation Point
Source: PLoS One. 2011 Jan 25;6(1):e16362. doi: 10.1371/journal.pone.0016362 (PMC3026816; doi:10.1371/journal.pone.0016362)
Supplement: Table S2 — (DOC) [file pone.0016362.s003.doc]

***Supplementary Table 2***

|  | Wild type | ΔGDH | ΔGOGAT |
| --- | --- | --- | --- |
| Doubling time (min) | 58 | 56 | 57 |
| J_1_ (mM/min) | 20.61871924 | 0 | 58.24060149 |
| J_2_ | 54.00000000 | 77.28367351 | 17.68721805 |
| J_3_ | 36.61773398 | 59.28061226 | 0 |
| J_4_ | 49.70985223 | 51.48520410 | 50.58195489 |
| J_5_ | 15.53497537 | 16.08979591 | 15.80751880 |
| J_6_ | 5.679310346 | 5.882142858 | 5.778947370 |
| J_7_ | 1.847290641 | 1.913265306 | 1.879699248 |
