# Supplementary material for: Flux Balance Analysis of Ammonia Assimilation Network in E. coli Predicts Preferred Regulation Point
Source: PLoS One. 2011 Jan 25;6(1):e16362. doi: 10.1371/journal.pone.0016362 (PMC3026816; doi:10.1371/journal.pone.0016362)
Supplement: Table S3 — (DOC) [file pone.0016362.s004.doc]

***Supplementary Table 3***

| Parameter | Description | Value | Unit | Reference |
| --- | --- | --- | --- | --- |
| K_αKG_ |  | 0.32 | mM | [1] |
| K_NH4_ |  | 1.1 | mM | [1] |
| K_NADPH_ |  | 0.04 | mM | [1] |
| K_NADP_ |  | 0.042 | mM | [1] |
| K_eq_ |  | 1290 | mM^-1^ | [1] |
| K_Glu_ |  | 10 | mM | [1] |
| L_ATP_ |  | 0.35 | mM | [2] |
| L_Glu_ |  | 4.1 | mM | [2] |
| L_NH4_ |  | 0.1 | mM | [2] |
| L_ADP_ |  | 0.0585 | mM | [2] |
| L_P_ |  | 3.7 | mM | [2] |
| L_Gln_ |  | 5.65 | mM | [2] |
| L_eq_ |  | 460 |  | [2] |
| M_Gln_ |  | 0.175 | mM | [3] |
| M_αKG_ |  | 0.007 | mM | [3] |
| M_NADPH_ |  | 0.0015 | mM | [3] |
| M_Glu_ |  | 11 | mM | [3] |
| M_NADP_ |  | 0.0037 | mM | [3] |
| Metabolites |  |  |  |  |
| NADPH |  | 0.13 | mM | [4] |
| NADP |  | 0.0021 | mM | [4] |
| ATP |  | 1.5 | mM | [4] |
| ADP |  | 0.6 | mM | [4] |
| Pi | inorganic phosphate | 10 | mM | [4] |

**References**

1. Sakamoto N, Kotre AM, Savageau MA (1975) Glutamate dehydrogenase from Escherichia coli: purification and properties. J Bacteriol 124: 775-783.

2. Rhee SG, Chock PB, Stadtman ER (1989) Regulation of Escherichia coli glutamine synthetase. Adv Enzymol Relat Areas Mol Biol 62: 37-92.

3. Rendina AR, Orme-Johnson WH (1978) Glutamate synthase: on the kinetic mechanism of the enzyme from Escherichia coli W. Biochemistry 17: 5388-5393.

4. Bruggeman FJ, Boogerd FC, Westerhoff HV (2005) The multifarious short-term regulation of ammonium assimilation of Escherichia coli: dissection using an in silico replica. FEBS J 272: 1965-1985.
