# Supplementary material for: Flux Balance Analysis of Ammonia Assimilation Network in E. coli Predicts Preferred Regulation Point
Source: PLoS One. 2011 Jan 25;6(1):e16362. doi: 10.1371/journal.pone.0016362 (PMC3026816; doi:10.1371/journal.pone.0016362)
Supplement: File S1 — The ordinary differential equations describing the labeling process of the metabolites. (DOC) [file pone.0016362.s005.doc]

The ordinary differential equations describing the labeling process of the metabolites are:

where denotes the external labeled ammonium measured in the agarose plate after switching, kd=7 min^-1^ is used to fit the diffusion delay of the external NH_3_ in the experiments; and denote labeled internal NH_4_ and labeled glutamate; denotes the single-labeled glutamine with main-chain nitrogen atom labeled; denotes the single-labeled glutamine with side-chain nitrogen atom labeled; denotes the double-labeled glutamine; and denote the summed concentrations of labeled and unlabeled glutamine and glutamate; and all other terms without star denote the unlabeled molecules, respectively.
