# Supplementary material for: Flux Balance Analysis of Ammonia Assimilation Network in E. coli Predicts Preferred Regulation Point
Source: PLoS One. 2011 Jan 25;6(1):e16362. doi: 10.1371/journal.pone.0016362 (PMC3026816; doi:10.1371/journal.pone.0016362)
Supplement: File S2 — Predicted key enzyme activities for ΔGDH and ΔGOGAT Strains. (DOC) [file pone.0016362.s006.doc]

***Predict Key Enzyme Activities for ΔGDH and ΔGOGAT Strains***

To compare with the western blots shown in Fig. 2C of Yuan’s work [1], we need to re-calculate the flux distribution of the nitrogen assimilation network under the same condition as in the experiments. First, we examined the comparability of cell growth for wild type, ΔGDH and ΔGOGAT strains. Based on their cell growth data (Figure 2a and Supplementary Figure 2 in [1], we roughly considered that with the same initial ammonium concentration (2mM or 10 mM), the wild type, ΔGDH and ΔGOGAT strains grew in a similar way. Second, we tried to estimate the growth rates for cells growing with 2mM initial ammonium. The cells obviously grew in a faster exponential phase before 3 hour (doubling time about 57 min) and in a slower phase (doubling time about 220 min) after 3 hour. However, the growth rate and doubling time at 3 hour were hard to identify, because this time point was just the turning point of growing phase from about 57 min to 220 min. To keep our model as simple as possible, we roughly estimated the doubling time at that moment as 110 min, which facilitated us to calculate the fluxes. So far, we obtained , and to calculate the V_max_ values of GDH, GS and GOGAT. Finally, we calculated the concentration of NH_4_^+^in based on the experimental external ammonium concentration. When the samples were taken after 3 hour grown in 10 mM initial ammonium, at the same time the measured total ammonium (NH_4_^+^ex + NH_3_ex) didn’t change so much (personal communication with Dr. Yuan). When the samples were taken after 3 hour grown in 2 mM initial ammonium, at the same time the measured total ammonium (NH_4_^+^ex + NH_3_ex) decreased to 0.75 mM taken from the Supplementary Excel file of Yuan’s work [1]. Substituted the concentrations of NH_4_^+^in, Glu, Gln and αKG (Supplementary Excel file in [1]) into Eq. 3, our model predicted V_max_ values of three key enzymes (shown in Table 2).

**References**

1. Yuan J, Doucette CD, Fowler WU, Feng XJ, Piazza M, et al. (2009) Metabolomics-driven quantitative analysis of ammonia assimilation in E. coli. Mol Syst Biol 5: 302.
